# From Protein to Pandemic: The Transdisciplinary Approach Needed to Prevent Spillover and the Next Pandemic

**DOI:** 10.3390/v13071298

**Published:** 2021-07-02

**Authors:** Raina K. Plowright, Peter J. Hudson

**Affiliations:** 1 Department of Microbiology and Immunology, Montana State University, Bozeman, MT 59717, USA; 2 Center for Infectious Disease Dynamics, Department of Biology, Pennsylvania State University, State College, PA 16802, USA; pjh18@psu.edu

**Keywords:** zoonotic spillover, bat viruses, henipaviruses, Hendra virus, Nipah virus, transdisciplinary research, pandemic prevention, pandemic origin

## Abstract

Pandemics are a consequence of a series of processes that span scales from viral biology at 10^−9^ m to global transmission at 10^6^ m. The pathogen passes from one host species to another through a sequence of events that starts with an infected reservoir host and entails interspecific contact, innate immune responses, receptor protein structure within the potential host, and the global spread of the novel pathogen through the naive host population. Each event presents a potential barrier to the onward passage of the virus and should be characterized with an integrated transdisciplinary approach. Epidemic control is based on the prevention of exposure, infection, and disease. However, the ultimate pandemic prevention is prevention of the spillover event itself. Here, we focus on the potential for preventing the spillover of henipaviruses, a group of viruses derived from bats that frequently cross species barriers, incur high human mortality, and are transmitted among humans via stuttering chains. We outline the transdisciplinary approach needed to prevent the spillover process and, therefore, future pandemics.

## 1. Transdisciplinary Science to Identify Drivers of Pandemics

Recent pandemics have been a consequence of viral spillover, where a pathogen passes from an animal host to a human through a series of processes that span scales from viral protein structure at 10^−9^ m to global transmission at 10^6^ m. The hierarchical sequence of spillover starts with an infected reservoir host shedding the pathogen, contact with a recipient host, infection of the recipient host, and then onward transmission within the recipient host population [1,2,3,4]. This process has been summarized as the infect, shed, spill, spread cascade [2]. The understanding and prevention of spillover require multiple scientific skills with expertise ranging from viral protein structure and adaptation to immunology, behavior, and physiology of the reservoir hosts, to interspecific contact, human behavior, and public health, shaped through national and international policy. 

Traditional approaches to the control of infectious diseases have centered on prevention, for example, quarantine and masks to prevent exposure, vaccines to prevent infection or disease, and therapeutics to prevent the progression of disease. These strategies work well against many human infections but are applied only after a pathogen has spread through the human population. We neither have the tool set to prevent the spillover of novel pathogens before they enter the human population, nor the rapid response mechanisms to restrict the spread of infection after spillover has occurred. This is a significant failure given the large impact on human mortality and suffering and the global financial cost of the ongoing COVID-19 pandemic [5]. This is also surprising given the 20 years of prior warnings of a future pandemic [6,7,8] and the accelerating rate of emergence of novel diseases, portended by SARS-COV in 2003, MERS in 2012, and Ebola in 2013–2016 [4,9,10]. In this paper, we address the following specific question: How do we prevent the next pandemic at the level of the reservoir host, before the pathogen has invaded the human host?

**Figure 1:**
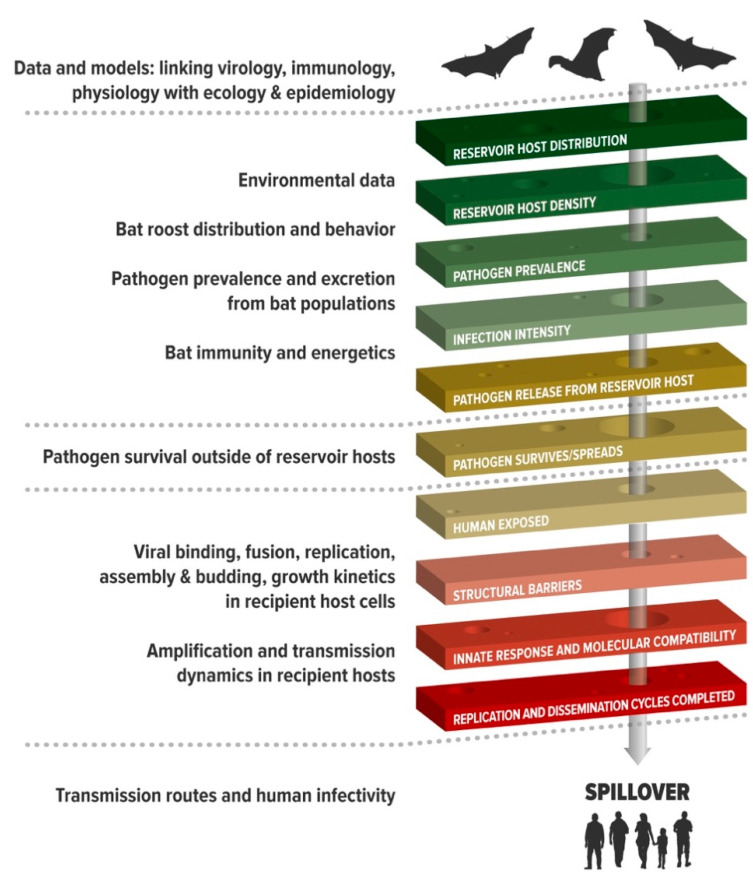
Biological data needed to understand and predict spillover (**left**) aligned to the key mechanisms of spillover (**right**). In addition, epidemiology and social sciences are employed to understand human exposure. Adapted from Plowright et al. [1].

Zoonotic spillover is the process of pathogen passage whereby the pathogen flows from a reservoir host to infect a human [1]. If the pathogen can efficiently replicate and transmit in the naive human hosts, human-to-human transmission may occur. Sometimes, adaptation in an intermediate bridging host is an essential step in the process to overcome host barriers [3,11,12]. For example, canine parvovirus increased its host range after circulating in raccoons [13] and simian immunodeficiency virus (SIV) evolved from an old-world monkey virus to become human immunodeficiency virus (HIV) after infecting chimpanzees or gorillas [14]. If the virus can transmit efficiently so that the value of the basic reproductive number (*R_0_*) is greater than unity, the new virus may continue to spread [4]. The whole process is a hierarchy with a set of specific barriers the pathogen must pass through to succeed in transmission in humans, and if the pathogen fails at any stage, onward progression is prevented [1] (Figure 1). For example, spillover requires the coincidental presence of reservoir and recipient hosts, and a pathway of exposure that will carry an infectious dose of pathogen to humans. If this is a virus, it must be able to counteract anti-viral factors at host mucosal surfaces, bind to host cell receptors, evade host innate immune responses, and replicate in tissues that allow passage out of the host for successful onward transmission [1,3,11]. Such alignment requires both suitable biological conditions and the coincidence of infectious virus with susceptible hosts.

Understanding the conditions that align this hierarchy requires deconstructing spillover into its component processes, and integrating data, models, and biological insights that explain spillover as an emergent property of a network of interactions, many of which are non-linear (Figure 1). Such research requires investigations that work at multiple scales. Consequently, this requires teams of transdisciplinary scientists who can identify the critical barriers and integrate their insights into models that explain patterns observed, and identify effective interventions. While this primarily requires biological insights, it transcends traditional biological disciplines to embrace environmental, computational, social, engineering, and health sciences, as well as the humanities, such as human geography, demography, religion, law, economics, and politics. Critically, the complexities and non-linearities in the spillover process need to be integrated through mathematical modeling or artificial intelligence to identify the components of the emergent network [15,16,17,18,19].

Creating and sustaining such transdisciplinary teams is not simple given issues of disciplinary semantics and the various perspectives of looking at complex systems. Solutions require frequent and intensive convening and coordination by leaders with an understanding of how the different members of the expert community view and solve problems, while creating unity through shared intellectual frameworks [20]. Teams must include scientists who are dedicated to solving complex, transdisciplinary problems and who are able to conceptualize and track system dynamics from molecular to global scales, and can apply knowledge, methods, and expertise outside of disciplinary boundaries. Team members who are more specialized must be both deeply versed in their disciplines, yet compelled to, and capable of, collaborating across disciplines. The members of these teams must be willing to contribute their time and patience, sometimes with few incentives or rewards from their institutions. 

## 2. The Next Pandemic: Preventing Spillover Is the Ultimate Approach 

If history is a predictor of future events, then the next pandemic will likely be a respiratory viral infection that emerges from an animal [21], probably a mammal and most likely a rodent or a bat [22]. The virus could be another unknown coronavirus, a henipavirus, a paramyxovirus, or an influenza virus. Infections from vector-borne diseases, such as Zika virus or West Nile virus, are unlikely to become pandemics since spread is limited by vector distribution and biting traits [23]. Bacteria rarely emerge following a jump into novel hosts, despite their ability to recombine and acquire novel genetic elements [24]; however, antimicrobial-resistant bacteria threaten rapid spread with no simple means of control and prevention [25,26]. A major fear is the emergence of a virus with high transmission efficiency and high mortality in some groups [4]—for example, a SARS-CoV-2-like virus with pre-symptomatic or asymptomatic transmission, a higher case fatality rate, and an *R_0_* greater than 4 could be cataclysmic. It is likely that such a virus is circulating in the wild. How can we prevent this virus from jumping into the human population and spreading? Can the next pandemic be stopped at the level of the reservoir host, before the spillover event?

When spillover leads to an outbreak in humans, the public health sector, appropriately, focuses on mitigating a public health emergency. Often, there is a lag between the original spillover and identification of the zoonotic pathogen in the human population, and this inhibits timely investigation of the spillover. For example, an investigative team did not arrive in Meliandou, Guinea, to examine the origins of the 2013–2016 Ebola outbreak in West Africa until ~four months after the spillover that triggered the epidemic [27]. By that time, the conditions that precipitated the spillover almost surely had passed. Similarly, the SARS-CoV-2 pandemic was investigated by an international team nearly a year after the pandemic started in China [28]. The thousands of infections and deaths from Ebola, and the millions of infections and deaths from SARS-CoV-2, were potentially triggered by a single spillover each. Most international attention and funding have not been focused on these events per se, but on cataloging the pathogens circulating in nature prior to such an event [29,30,31] or, more critically, on detecting pathogens in high-risk animal and human communities after such an event [32,33]. Preventing spillover would surely be the ultimate pandemic prevention, but this would require focusing on the multiple processes that drive the spillover event. Developing a framework for such an approach to pandemic prevention is most feasible in systems where spillover is common, and research can focus on multiple past spillover events. 

## 3. Systems with Replicated Spillover Events Provide Insights into the Processes

Understanding the processes of spillover requires detailed data from replicated spillover events. As such, many of the pathogens of concern are not good candidates for developing the insights needed for spillover prevention. For example, SARS-CoV-1, SARS-CoV-2, and most Ebola outbreaks may have originated from a single spillover event from bats (usually to a bridging host before reaching humans). Therefore, the sample size for studying spillover events is small. Moreover, since the reservoir hosts of these pathogens have not been identified, the drivers of spillover are difficult to study [34]. With more investment and investigation, it is likely that SARS-like beta-coronaviruses (sarbecoviruses) that regularly spill over to humans, perhaps with mild disease and limited onward transmission, will be identified soon [35,36]. These viruses should become testbeds for developing an understanding of spillover of pathogenic sarbecoviruses. Currently, Hendra virus and Nipah virus, two of five characterized henipaviruses, appear to be the most promising candidate systems for studying the spillover of bat-borne viruses because, compared to other bat-borne viruses, spillovers are detected relatively frequently.

**Figure 2:**
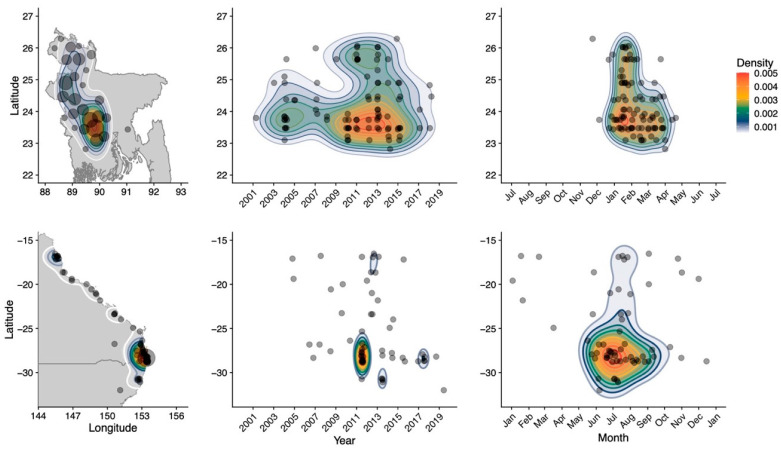
Number of Nipah virus (**top**) and Hendra virus (**bottom**) spillovers by latitude and longitude (**left**), latitude and year (**middle**), and latitude and month (**right**). Top panel adapted from McKee et al. [37] (this issue).

In Bangladesh, multiple spillovers of Nipah virus have been detected regularly since 2001 [37,38], and in subtropical Australia, clusters of spillovers of Hendra virus have occurred every 2–3 years over the past decade and single spillovers of Hendra virus are detected almost annually [34] (Figure 2). Since human-to-human transmission is rare for Nipah virus and absent for Hendra virus, each one of these records appears as an independent spillover event. In both countries, most spillovers occur during winter [34,37], and the multiple annual spillovers allow spatial and temporal patterns to be observed and studied (Figure 2).

Most of the recent human infections with Nipah virus were caused by the consumption of date palm sap after bats contaminated the sap through urine or saliva [39]. Hendra transmission to humans is a consequence of exposure to a bridging host, which is an infected horse. In both instances, the route of transmission may result in human exposure to a large dose of virus that subsequently causes a severe infection. If there were horses in Bangladesh or if date palm sap was consumed in Australia, the transmission routes for the viruses might be interchanged, but both routes may lead to a high dose of human exposure.

Although there is evidence of direct human-to-human transmission of Nipah virus, the chain of transmission rapidly breaks down, probably because only 9% of cases transmit [38]. As a human infected with Nipah virus generally does not infect more than one other person, the basic reproduction number, R_0_, is <1, so the virus does not spread widely in human populations [38]. Therefore, the identification of cases in humans implies that the spillover occurred relatively recently, and it may still be possible to identify the conditions that aligned to allow the virus to flow across species.

## 4. Transdisciplinary Approach to Stop Spillover

**Figure 3:**
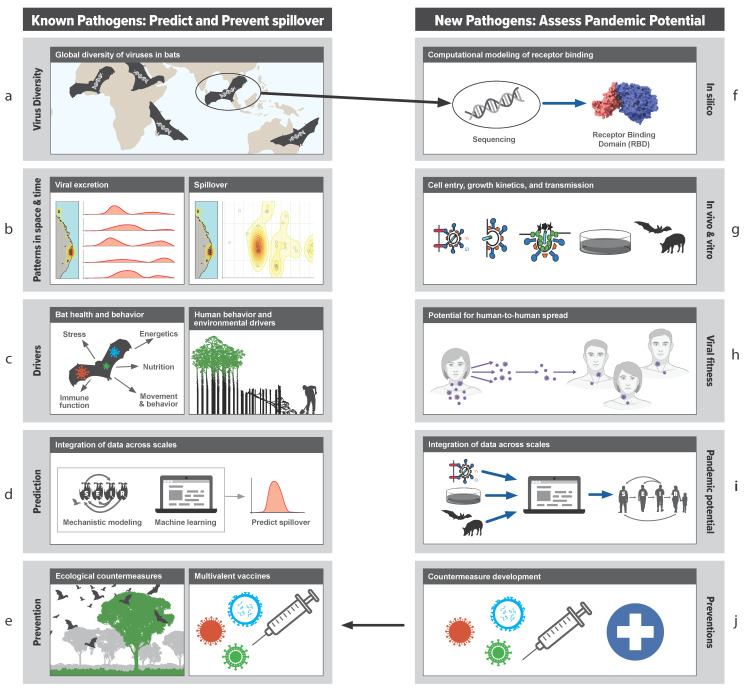
Transdisciplinary and convergent approach to studying pathogen spillover by Bat One Health (www.batonehealth.org, accessed on 25 June 2021). On the left side, ecological, physiological, and behavioral information is collected alongside host–viral dynamics (**a**–**c**). Data are integrated within models to make predictions (**d**) and to develop interventions to prevent spillover (**e**). On the right is a proactive means to assess pandemic potential of viruses by examining viral fitness in silico, in vitro, and in vivo (**f**–**j**).

As a worked example of the transdisciplinary approach to studying bat virus spillover, we now examine the approach taken by the Bat One Health team (www.batonehealth.org, accessed on 25 June 2021) that have used henipaviruses to develop a framework for understanding and preventing spillover (Figure 1 and Figure 3). By studying each component of the spillover hierarchy (Figure 1), they identified the key processes that drive spillover, formed transdisciplinary teams to study components in detail, and then integrated the information across multiple levels (Figure 3). The following sections describe the key research areas that we think are critical for pandemic prevention. Using such an approach, we propose that the data, models, and insights developed in the henipavirus systems could be considered a framework of understanding that could be applied to other pathogens with less tractable spillover events, such as the sarbecoviruses and ebolaviruses.

### 4.1. Ecology of the Reservoir Host

In 1973, Theodosius Dobzhansky famously stated that “nothing in biology makes sense except in the light of evolution” [40]. We suggest that for emerging infectious diseases, nothing makes sense except in the light of the ecology of the reservoir host. The events that trigger spillover are most likely to be rooted in environmental changes that fundamentally alter host ecology, including host distribution, population dynamics, feeding behavior, condition, and consequently, host-pathogen dynamics [2].

Essentially, the risk of spillover in space and time is a function of the distribution of a pathogen across a landscape, which is in turn determined by the distribution of the reservoir host species [41] (Figure 3a). However, some reservoir host populations may present a higher spillover risk than others. Many bat species are sensitive to land-use changes that disturb their feeding or roosting habitats [42,43]. To survive these stressors, some species adapt via emergency life-history strategies in which they exploit suboptimal, human-dominated environments [43,44]. The consequence is greater contact with humans and higher levels of pathogen shedding. For example, when flowering does not occur or flowering trees are removed, nomadic nectivorous bats become resident frugivores in urban areas to reduce the energetic cost of foraging [45,46,47]. The risk of spillover from these populations is high because they have regular contact with humans and are energetically stressed and more likely to shed virus [48]. As an illustration, in response to the loss of winter habitat, Australian flying foxes rapidly relocated to urban and agricultural areas coincident with the emergence of Hendra virus [34]. By contrast, in Bangladesh, the transition of *P. medius* to urban residency probably occurred decades ago due to historic deforestation [37]. 

Sampling to assess spillover risk should focus on populations experiencing changes in distribution and environmental stress, and populations that are behaving abnormally (e.g., feeding on low-quality food in human landscapes). Remote sensing and AI prediction can help to locate these populations, but long-term population surveys are essential to document how bat populations, and the risk of spillover, are changing in response to anthropogenic stressors. 

### 4.2. Viral Shedding from Reservoir Hosts and Its Drivers

Many pathogens, including the bat henipaviruses, coronaviruses, and filoviruses, have dynamic and fluctuating relationships with their hosts [49] (Figure 3b). Infection and shedding can be seasonal and driven by cycles of breeding, aggregation, and the introduction of naïve young individuals into the host population [50,51,52]. Moreover, age, sex, breeding status, nutrition, coinfections, and stress contribute to variation among individuals such that 20% of hosts may be responsible for 80% of transmission events [53,54]. The reactivation of latent virus in bats during stressful periods has also been proposed as a driver of henipavirus shedding [51]. The simultaneous excretion of multiple paramyxoviruses along with Hendra virus, in brief, spatially restricted pulses coincident with nutritional stress, is further evidence that periods of stress may affect viral excretion from bats [55].

Identifying times and places in which the risk of spillover is elevated requires repeated sampling of reservoir host populations in space and time [41,49] (Figure 3b). By contrast, cross-sectional sampling is more likely to identify pathogens that chronically infect the host; pathogens that cause transient infections or intermittent shedding such as filoviruses and paramyxoviruses may be missed altogether [49]. Optimal sampling methods, such as adaptive pooled sampling, Bayesian data integration, and model-guided sampling, may help determine the most efficient sampling frequency, intensity, and extent [18,56,57]. Metadata on host demography, condition, and immunity is critical to understanding how environmental stressors affect pathogen shedding [58,59], but a lack of reagents [60] and poor knowledge of bats’ novel antiviral defenses [61] impede these studies. To establish causal inference, the correlative interpretation of field observations should be complemented by experiments that replicate the putative causal stressor [62]. For example, treatment groups may be deprived of certain nutrients and then challenged with viral-like particles, pseudoviruses, or real viruses. A controlled experiment may minimize confounding variables but will also miss the emergent properties and interactions in the ecological system. Therefore, field experiments where systems are perturbed, and outcomes are compared to model predictions, could prove insightful.

### 4.3. Bat and Human Behavior and Proximity

The ecological and virological factors discussed above generate pathogen pressure, but the behavior of both reservoir hosts and humans enables the delivery of an infectious dose of virus through an effective route of exposure. For example, the *Pteropus medius* (Indian flying fox) feeds on date palm sap, and humans consume this sap; the *P. alecto* (black flying fox) feeds in horse paddocks; horses consume contaminated grass; and humans are exposed through veterinary interventions, such as endoscopy of a horse in respiratory distress [39,63]. Intensive epidemiological and sociological investigations are required to understand these mechanisms, and the pathways to zoonotic spillover for many systems remain a mystery. For example, the conditions that led to Nipah virus spillover in Kerala, India; Ebola virus spillover in West Africa; and SARS-CoV-2 spillover in Asia are still unknown [27,28,64]. It is challenging to identify the infected reservoir hosts responsible for spillover (e.g., bats that feed on date palm sap), and the humans that are becoming exposed (e.g., immunocompromised individuals, such as HIV-positive persons).

Human perceptions of spillover risk and reactions to the risk of infection are complex [65]. Clear, distinct narratives are prevalent in different groups within a community that perceive the risk in different ways. These narratives are rarely based on knowledge and often based on viewpoints of peer groups and misconceptions [65]. For example, Australians may respond negatively to large bat colonies that appear in urban areas to feed on nearby, ephemeral nectar pulses. The excreta from bats in these roosts damages property, and the smell is unpleasant. However, the risk of direct spillover to humans from these bats is low. Indeed, most spillover events to date have occurred in agricultural areas recently occupied by bats, and by bats feeding on poor-quality fruit rather than high-quality nectar [34], and all cases involved a horse as a bridging host [66]. Fortunately, there is an effective horse vaccine for Hendra virus [67,68], but uptake by horse owners has been low. By contrast, in Bangladesh, awareness of the risk of infection from date palm sap has not stopped the consumption of the sap but has led to simple interventions, such as covering collection pots to prevent bat contact with the sap [69]. Within China, although it was well known that the spillover of SARS-CoV-1 occurred within a wet market [70], the risk of exposure to a second sarbecovirus effectively was ignored by policy makers; breeding of wild animals for food and the importation of bushmeat and live animals continued. Including behavioral sciences in the integrated approach to pandemic prevention allows the behaviors that drive pathogen spillover to be identified and investigated [71]. 

### 4.4. Data Integration

With any transdisciplinary study, the challenge is to integrate the life and social sciences in a manner which captures the key drivers, explains previous patterns, and predicts future events (3d). For spillover, small changes in one part of the system may drive large changes in risk. For example, infected bats sharing date palm sap with susceptible humans may be widespread across a landscape, but small surges in the pathogen load in bats, or small increases in the sap drinking behavior of humans, may tip the system into spillover. Thus, spillover is an emergent property of non-linear interactions, from host density to infectious dose effects [1,72]. Integrative approaches, such as mechanistic modeling, machine learning, and simulation, are needed to integrate data to understand these non-linear interactions, develop predictions, and identify points of intervention [58,64,73,74,75]. Once the drivers and dynamics are understood, it becomes more feasible to devise interventions that will work, for example, ecological countermeasures that address the root cause of emergence (e.g., modification of land cover [76]) or medical countermeasures that can be deployed in response to predicted spillover events.

### 4.5. Functional Characterization of Viruses

Bats harbor a diverse array of viruses, including many variants of henipaviruses, filoviruses, coronaviruses, and paramyxoviruses, many of which have unknown zoonotic potential [22,77]. Identifying new viruses can be useful for understanding the natural history of host–viral interactions and natural diversity. However, most viruses cause no harm to the reservoir host or new recipient hosts. Where there is an abundant diversity of viruses, the probabilistic outcome of each contact between the virus and recipient host reflects whether the virus passes through the series of barriers to establish an infection. Therefore, we recommend that viral discovery be combined with the intensive functional assessment of the ability of each virus to pass through these barriers (Figure 3f–j).

Ideally, bats should be sampled across a wide geographic range, including in novel habitats and high-risk interfaces (Figure 3a) to identify new variants for the characterization of zoonotic potential [30]. In silico estimates of binding to human receptors can be followed with in vitro and in vivo studies of virus entry, replication, assembly, budding, fusion, and growth kinetics [78,79,80,81]. After the potential for human infection is estimated, the potential for human-to-human transmission, and therefore, pandemic potential, can be assessed via in vivo experiments in model species [82]. The integration of virology and epidemiology through modeling is essential to ensure that virological data can be scaled up to generate epidemiological insights [83]. This connection from genotype to phenotype builds a knowledge base for risk assessment. Variants of concern can be prioritized for detailed studies (Figure 3a–d) and for the development of countermeasures (e.g., multivalent vaccines and therapeutics; Figure 3e).

The research agenda we propose (Figure 1 and Figure 3) will help transition the pandemic response from the post hoc reaction after the pandemic has spread in human communities to proactive arresting of the pandemic process before spillover occurs. Our transdisciplinary emphasis facilitates identification of the risk of viral spillover in space and time and the mechanisms driving this risk, which in turn allows for a focus on addressing the root causes of viral emergence or intervening at each stage of the spillover process. If all efforts to prevent the spillover fail, accurate predictions at levels that are actionable for public health intervention allow the deployment of vaccines and therapeutics for virus genotypes that pose the greatest risks, before an outbreak escalates into a pandemic. However, the ultimate pandemic prevention is prevention of the spillover event itself.
